# Surgical treatment of advanced penile cancer

**DOI:** 10.1007/s00432-017-2435-1

**Published:** 2017-05-10

**Authors:** Ke Zhang, Xiang Wan, Huan Xu, Wenzhi Li, Juan Zhou, Ming-Xi Xu, Hai-Jun Yao, Zhong Wang

**Affiliations:** Department of Urology, Shanghai Ninth People’s Hospital, Shanghai Jiao Tong University School of Medicine, No.639 of Zhizaoju Road in Huangpu District, Shanghai, 200011 China

**Keywords:** Advanced, Penile cancer, Therapy, Surgery

## Abstract

**Purpose:**

To evaluate the therapeutic effect of surgery in patients with advanced penile cancer, who have a dismal prognosis.

**Patients and methods:**

Between September 2007 and July 2015, we treated 12 patients with surgical therapy.

**Results:**

The median follow-up period for all the patients was 16 months (range 4–60 months). The outcome and concomitant symptoms were analyzed, and the survival rates were calculated. Three of the patients are currently alive. The median overall survival of the patients was 9 months (range 4–13 months).

**Conclusion:**

The present results suggest that surgery is a valuable treatment option for patients with advanced penile cancer. The prognosis of advanced penile cancer is closely related to lymph node and distant metastases. Flap repair can solve the problem of large area skin defect. Advanced penile cancer is difficult to treat regardless of chemotherapy or radiotherapy, and surgery cannot prolong the lives of patients. However, the dissection of lesions and repair of large area skin defects can dramatically improve the quality of life of patients, especially those with locally advanced disease without distant metastasis.

## Introduction

Penile cancer (PC) is an uncommon malignant tumor, with around 4000 cases diagnosed each year, accounting for less than 0.5% of all cancers (Mosconi et al. 2005). It is rare in Western countries, but not in developing countries (Micali et al. 2006). Unfortunately, its incidence continues to increase in parts of Asia, Africa, and South America (Ornellas 2008; Misra et al. 2004), but decreased in the United States from 1973 to 2002. In 2012, 1570 new cases and 370 deaths were documented in America (Siegel et al. 2012; Barnholtz-Sloan et al. 2007). In less developed nations, the condition is even worse, such as in sub-Saharan Africa and parts of South America, where it accounts for around 10% of all male malignancies (Heinlen et al. 2012).

The focus of this article is advanced penile cancer which can be defined as bulky lymph node metastases (cN2 or cN3), failed primary lymphadenectomy leading progression to nodal metastases, matted or bulky lymphadenopathy (cN3), 2 metastatic superficial and deep inguinal lymph nodes (pN2), metastatic pelvic lymph node (N3), local erosion to pubic bone, abdominal wall, or pelvis (T4), and distant metastases (M1) (Heinlen et al. 2012).

The current therapies for advanced penile carcinoma include surgical therapy, radiotherapy, chemotherapy, or multimodality therapy (Heinlen et al. 2012). The prognosis in advanced penile carcinoma is poor, especially in patients with more than 2 inguinal lymph node metastases. The 5-year survival rate of these patients is 7–50%. Mortality markedly increases when pelvic lymph node metastasis develops, which means a 5-year survival of <5% (Srinivas et al. 1987; Ravi 1993). We report our experience of using surgical treatment in the management of patients with advanced penile cancer.

## Patients and methods

After Institutional Review Board (IRB) approval, we identified 12 patients who underwent surgical treatment for advanced penile cancer at our hospital. We retrospectively evaluated these patients who were treated between September 2007 and July 2015 in our hospital and followed up until December 2015. Five patients had ECOG performance status 2; five patients had ECOG performance status 3; two patients had ECOG performance status 4 before the operation.

**Fig. 1:**
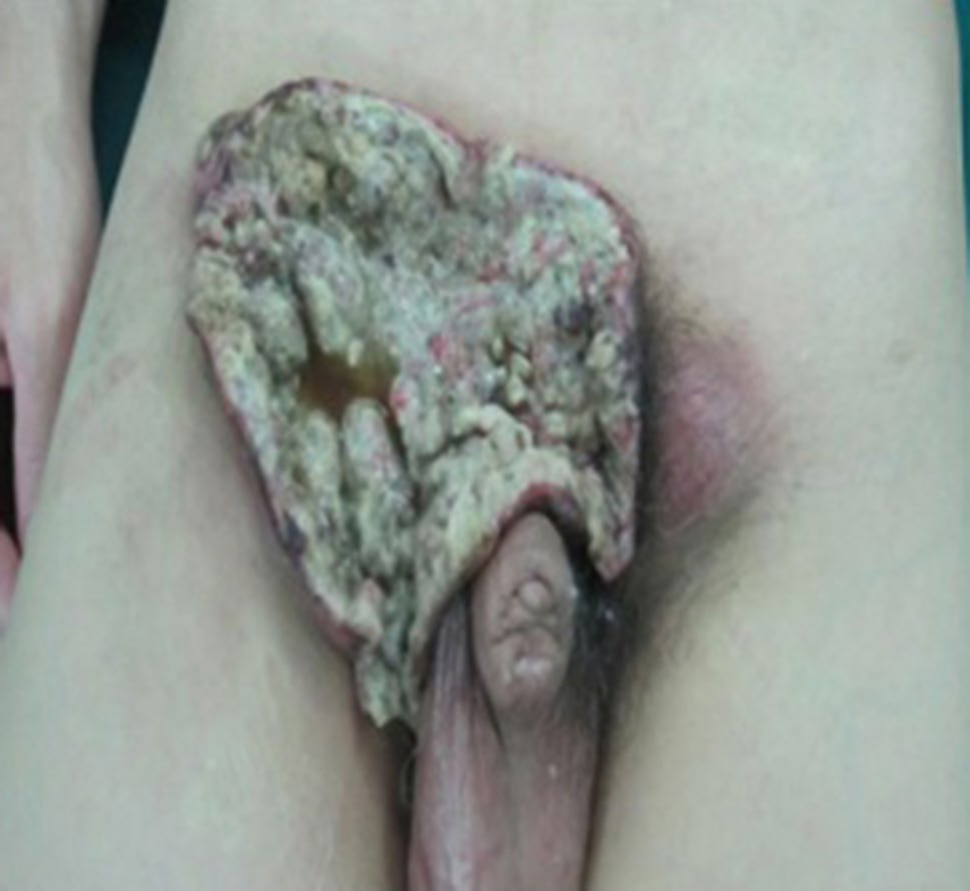
Tumor invades the right inguinal region, skin erosion with pain

**Fig. 2:**
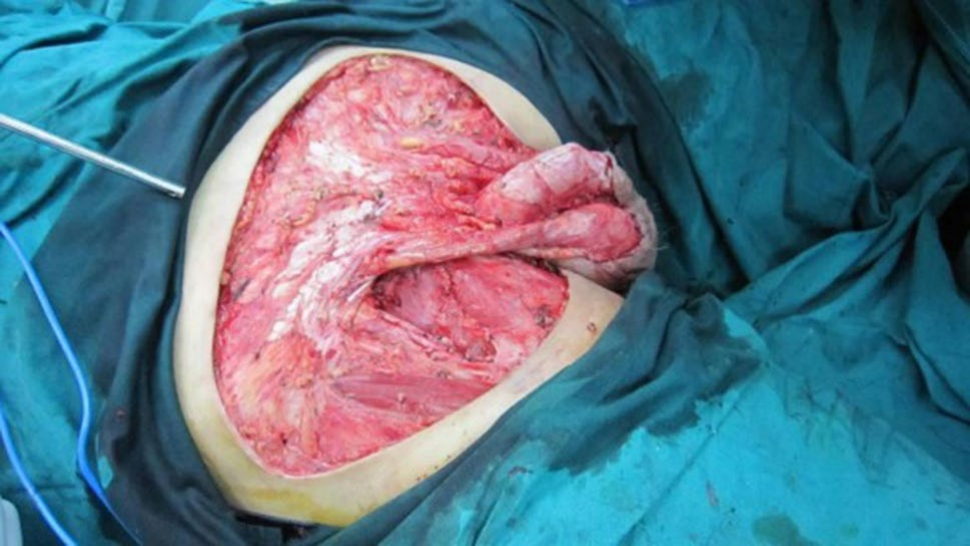
The defect after the tumor resection

**Fig. 3:**
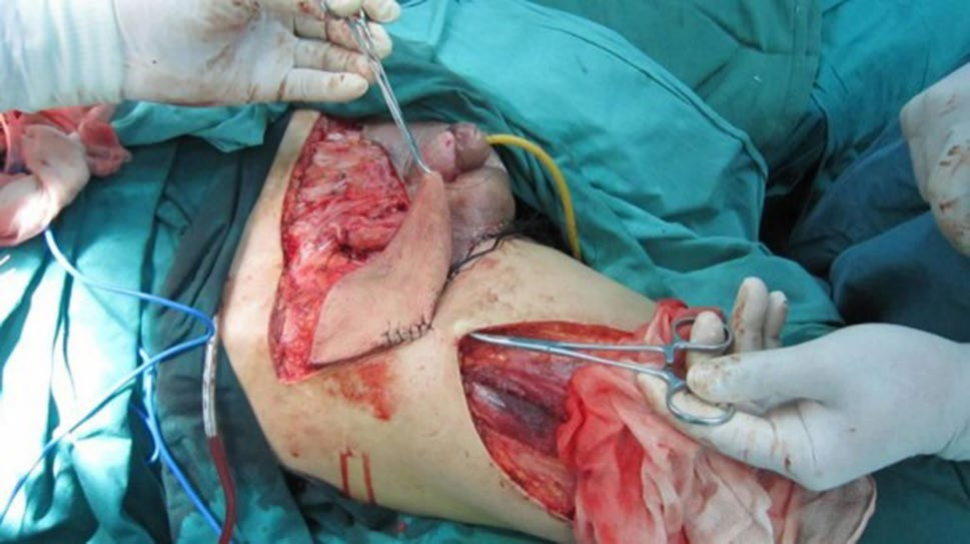
We use right side of the lateral femoral circumflex perforator flaps cover the defect

All of the 12 patients received partial penectomy or radical penectomy with inguinal lymph node dissection (Table 1). Patients of No. 1, 2, 5, 12 received abdominal wall defect repair and skin grafting because of the large area skin defects (Figs. 1, 2, 3). Two patients received postoperative chemotherapy (ifosfamide, paclitaxel, and cisplatin), and one patient received radiotherapy (50 Gy).

**Table 1:** Surgery and adjuvant therapy of 12 patients

Patients	Surgery on penis	Using ALT flap	Adjuvant therapy postoperatively
1	Total penile resection	Yes	No
2	Total penile resection	Yes	No
3	Partial penectomy	No	No
4	Total penile resection	No	No
5	Total penile resection	Yes	No
6	Total penile resection	No	No
7	Partial penectomy	No	Chemotherapy + radiation
8	Total penile resection	No	No
9	Partial penectomy	No	No
10	Total penile resection	No	Chemotherapy
11	Partial penectomy	No	No
12	Partial penectomy	Yes	No

## Results

**Fig. 4:**
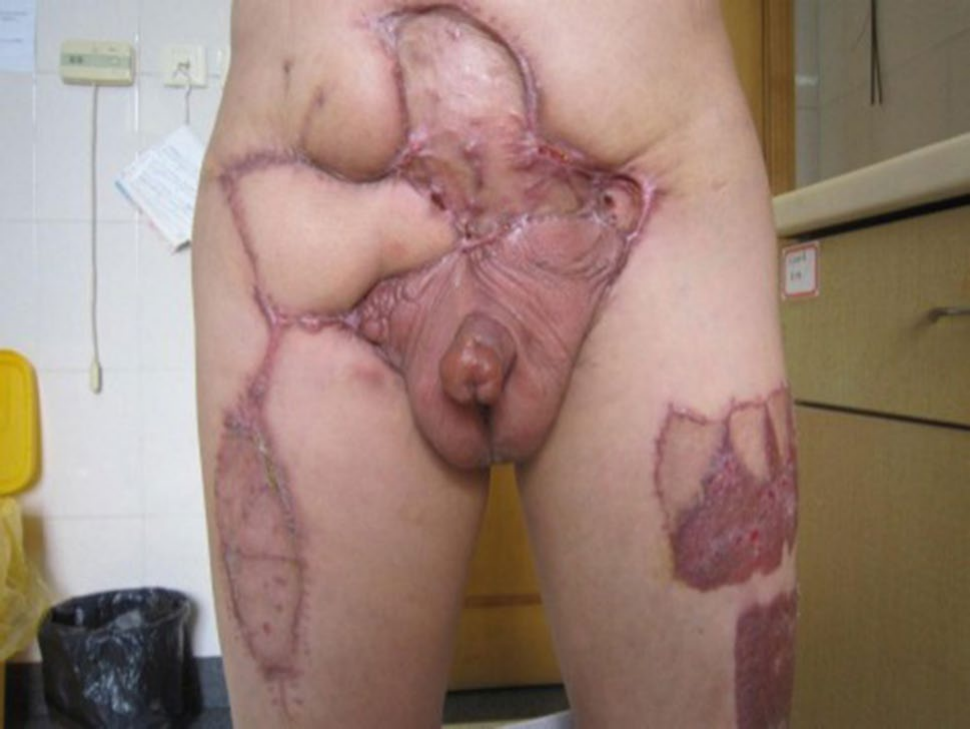
Flap and donor site healing well 3 months after the operation

The median patient age was 54.6 years (range 37–89 years). The clinical data of the patients are summarized in Table 2. The flap of the three patients with large area defects healed well (Fig. 4) and one patient delayed healing, two patients experienced edema of lower extremity. No severe complications occurred during hospitalization and 10 of 12 patients thought their dysuria, pain ameliorated, quality of life improved dramatically and the surgery rewarding. All the 12 patients were followed up 16 months on average (range 4–60 months), three patients are currently alive, and the other nine patients died (mortality, 75%) because of the progression of the original disease or distant metastases. The mean time from operation to death was 9 months (range 4–13 months). Three of the four patients with regional advanced PC without distant metastasis are currently alive, with 60 months as the longest period. We offer the palliative care to assess the multifaceted needs of patients to help them combat issues of pain, constipation, psychological and cognitive effects.

**Table 2:** Clinical characteristics of 12 patients with advanced penile cancer

Patients	Age	Grade	Stage	Tumor size (cm)	Lymph node metastasis	Distant metastasis	Concomitant symptoms	Symptom improve?	Survival (month)
1	37	G2/3	T4NOMO	20 × 20	No	No	Dysuria	Yes	12
2	67	G2	T4NOMO	10 × 12	No	No	Dysuria	Yes	Alive (60)
3	89	G2	T2N3M1	4 × 4	Bilateral inguinal region + pelvic capacity	Lung	Pain	Yes	10
4	55	G2/3	T2N3M0	4 × 3	Bilateral inguinal	No	No	No	4
5	40	G1	T4N0M0	8 × 15	No	No	Dysuria	Yes	Alive (31)
6	43	G1/2	T3N3M0	6 × 4	Bilateral inguinal region + pelvic capacity	No	Dysuria	Yes	11
7	52	G1	T4N2M0	8 × 12	Bilateral inguinal	No	Pain	Yes	Alive (18)
8	50	G2	T4N2M1	8 × 10	Bilateral inguinal	Lumbar	No	No	9
9	47	G3	T4N3M1	9 × 10	Bilateral inguinal region + pelvic capacity	Lung	Dysuria	Yes	5
10	60	G2/3	T3N2M1	7 × 4	Bilateral inguinal region	Lung	Dysuria	Yes	6
11	67	G2	T2N3M0	4 × 4	Right side inguinal region + pelvic capacity	No	Pain	Yes	5
12	48	G1/2	T2N3M0	15 × 12	Right side inguinal region	No	Pain	Yes	13

## Discussion

Treatment for advanced PC is still a worldwide problem, as the ideal prognosis of patients has not been achieved. The effects of radiotherapy and chemotherapy are not good. Radiotherapy can be performed as a single agent, but its usefulness is questionable. While data are scant, existing evidence shows the efficacy of chemotherapy for this disease. Administration of chemotherapy in addition to surgery or radiotherapy may increase patient survival (Heinlen et al. 2012). However, no consensus has been reached as to the significant efficacy of chemotherapy or multimodality therapy for advanced penile carcinoma or metastatic PC. New effective drugs should be developed, and multicenter cooperation is essential.

Advanced PC is associated with poor prognosis and is closely related to lymph node metastasis. The more lymph node metastases found, the worse the condition. Pandey et al. (2006) evaluated 102 patients with inguinal lymph node. Patients with 1–3, 4 or 5, or >5 lymph nodes involved have a 75.6, 8.4, or 0% 5-year survival rate, respectively. According to Yao Zhu et al. (2012), unilateral and bilateral lymph node metastases have 3-year recurrence-free survival rates of 59.2 and 26.7%, respectively. Much evidence has proven that the ratio of positive lymph nodes have more advantage than number-based nodal staging in predicting cancer prognosis (Vinh-Hung et al. 2009a, b; Berger et al. 2005). According to Svatek et al. (2009), the 5-year disease-specific survival rates in patients with lymph node ratios of <6.7 and >6.7% are 91.7 and 23.3%, respectively, which indicates a statistically significant difference. Pelvic lymph node involvement is an independent poor prognostic factor. Pandey et al. reported that none of their 21 patients with pelvic lymph nodes survived for even at least >3 years. Similar findings were reported by Ravi (1993). According to Zhu et al. (2012), patients with sentinel lymph nodes of <2 and >2 mm in size had disease-specific survival rates of 94.4 and 69.5%, respectively. The size of the sentinel lymph node can predict additional lymph node metastasis, but this needs further study. Among our 12 patients, 6 with N3 stage disease died with a 7.4-month survival on average. Their prognosis was poor, consistent with a previous report in the literature (Zhu and Ye 2012).

Obviously, distant metastasis is an important factor that affects prognosis. According to Bermejo et al. (2007), in their study, one patient with lung metastases who underwent BMP (bleomycin–methotrexate–cisplatin) chemotherapy only survived 7 months. Another patient had liver and lung metastases after 5 months of BMP treatment and died 2 months later. According to Carthon et al. (2014), 14 patients with distant metastases (M1) received epidermal growth factor receptor-targeted therapy, with a mean overall survival period of 264 days (range 31–1332 days). According to Di Lorenzo et al. (2009), three patients with distant metastases that included liver, lung, or abdominal lymph nodes had a median overall survival of 8 months. All of the four patients with distant metastases (3 lung metastasis and 1 lumbar vertebrae metastasis) died, with a mean survival of 7.5 months (range 5–10 months).

Patients with locally advanced disease without distant metastasis and N3 lymph node metastasis have a relatively long survival. Four of the 12 patients had T4N1-2M0 stage disease, 3 of whom are currently alive, but 1 died after 12 months. The longest survival period was >5 years. We should perform radical surgery for patients with partial advanced PC without clear metastasis if their physical conditions permit. For the seventh N category, the 3-year recurrence-free survival rates of N1, N2, and N3 were 87.5% (*n* = 16), 57% (*n* = 22), and 31.8% (*n* = 22), respectively (Zhu and Ye 2012). According to Liu et al. (2013), patients with T0-3N0-2M0 disease have an overall survival of >14 months, and the longest surviving patient is still alive up to now.

Some scholars put forward multimodality therapy according to the poor effect of the treatment of advanced PC. According to Komine et al. (2014), a patient (80-year-old, pT1pN0) who underwent penectomy and bilateral inguinal lymphadenectomy had a disease relapse after 1 year. He was treated with TPF (paclitaxel–cisplatin–5-FU) chemotherapy and 50-Gy external beam radiotherapy to the inguinal region, along with Mohs’ paste. For socioeconomic reasons, the patient canceled his treatment and showed no progression or metastasis after 8 months, but died after 12 months. According to Pagliaro et al. (2009), preoperative (neoadjuvant) combination chemotherapy can dramatically improve the progression-free survival of patients with lymph node metastases. Postoperative radiotherapy can be performed depending on the amount of residual disease. Chemoradiotherapy has been proven effective for unresectable PC. However, the number of cases treated with multimodalities is too small. No consensus has been reached as to the appropriate approach for multimodality therapy for advanced PC.

Although advanced PC is associated with poor prognosis, surgical treatment is still a relatively effective approach. Most of our 12 patients had dysuria or pain. Some of them had a large wound erosion area, which affected their quality of life. For these patients, control of local and systemic disease is the goal of treatment (Heinlen et al. 2012). All the 12 patients received surgical therapy, including partial penectomy or radical penile dissection and inguinal lymph node dissection, and 1 pelvic lymph node dissection. Our follow-up indicated that the postoperative symptom of dysuria and pain in these patients greatly improved.

Flap repair can solve the problem of skin defect after penectomy. Kayes et al. (2007) performed vertical rectus abdominis flap reconstruction in patients with advanced PC in 2007 and yielding a satisfying outcome. We performed thigh myocutaneous flap reconstruction or large-sized skin graft for four cases with large skin defects. The effect of the use of a myocutaneous flap was better because of abundant blood supply. It allowed for faster healing and reduced the risk of infection. The complete free flap has limited blood supply; thus, the healing time is longer. However, it is advantageous for surgical trauma. The flap can be chosen according to skin defect size, and physical and skin conditions. The four patients received a skin graft or flap reconstruction, and healed well and had greatly improved quality of life.

NCCN recommended T ≥2 patients require more extensive surgical intervention with partial or total penectomy to remove the lesions; Patients with palpable nodes advised standard or modified ILND (inguinal lymph node dissection); Patients with 2 or more positive ILNs (inguinal lymph node), poorly differentiated metastases, or extracapsular nodal extension are recommended to PLND (pelvic lymph node dissection) (Clark et al. 2013). We evaluated the age, physical status, primary tumor, inguinal lymph node metastases, and pelvic lymph node metastases of the 12 patients. We recommended neoadjuvant followed by surgical treatment, but for certain reasons they refused, so we performed surgery for them. All the 12 patients considered physical functioning, role functioning, emotional functioning, cognitive functioning, and social functioning improved significantly and deem the surgery rewarding. In addition to 1 flap delayed healing and 2 edema of lower extremity, all patients felt their symptoms alleviated allowing them a reasonable quality of life. No severe complications occurred postoperatively. So we think that we relieved their pain and improved their quality of life.

The low incidence and dispersed data of PC led to the lack of systematic comparison data. In addition, the classic paradigm of randomized trials may be difficult to execute for this rare malignancy (Sonpavde et al. 2013). The few reported cases led to data bias. All our 12 patients received surgical therapy. However, we could not compare our patients to those who did not undergo surgery because of the lack of detailed nonsurgical treatment data. Thus, we rely on foundations or international organizations for PC, like other cancers, to promote the communication and research between physicians to help patients with advanced PC. Moreover, a specific and detailed questionnaire should be developed to assessing quality of life for penile cancer.

## Conclusion

Advanced PC is difficult to treat regardless of chemotherapy or radiotherapy, and surgery cannot prolong the lives of patients. However, dissection of lesions and repair of large area skin defects can dramatically improve quality of life, especially, that of patients with locally advanced disease without distant metastasis. We recommend multimodality therapy, but in some cases surgery could be performed firstly to improve quality of life.
